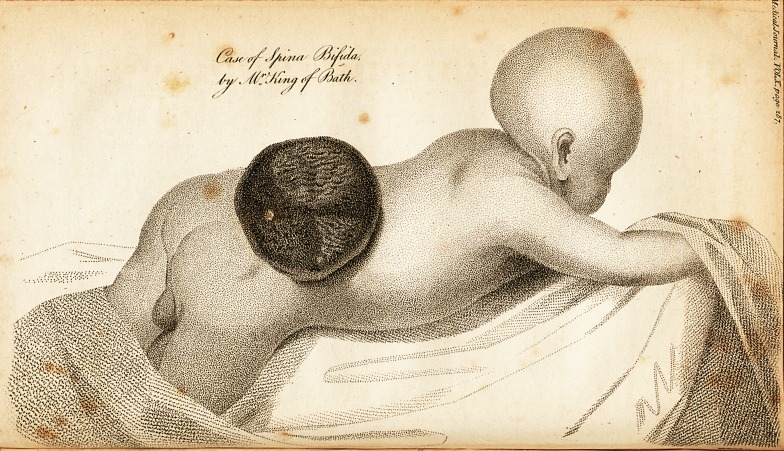# A Case of Spina Bifida

**Published:** 1803-09-01

**Authors:** John King

**Affiliations:** Bath


### A Case of Spina Bifida; *communicated by* Mr. John King, *of Bath.*

[ With an Engraving. ]

On the morning of the second of March last, I was called to a poor woman in Bath to attend her in labour, when I found the patient in the regular progress; the os uteri dilated, and the membranes not ruptured; the pains, or what is analogous, the contractions, frequent, though not strong or lasting; the os externum relaxed, and the pelvis appeared to be well formed. I encouraged the recurrence of pain by stimuli (as she had remained a long time in this situation without any signal advantage from the pains); in a few hours the child descended into the pelvis. The bag of waters became ruptured, yet I was not satisfied as to the presentation, and I soon found it was a breech case; for as the parts came to wedge in the pelvis, the scrotum of the child was squeezed out and swoln. On conducting my finger along the sacrum of the child, till I reached the lumber vertebræ, I found a considerable fossa in the loins, covered with a loose membrane, with the bones of the spine bifurcated; I then apprehended a delay to the farther progress of the labour, because it appeared probable that the diameter of the child's pelvis might be preternaturally enlarged, owing to the bony parietes formed by the division of the spine; but I determined in a well-formed pelvis not to turn, but to risk the extraction in statu quo. In this instance I never more strongly saw the good effects resulting from the plan I adopted in promoting the parturient exertions of the woman, by plying her repeatedly with brandy and water, without which I could have had no farther hopes of delivering the patient, unless by turning, which I wished to avoid; but I would have the utmost caution used in such cases, as their presentation offers no probability of delivery, livery, or where the presentation is not clearly ascertained, as it would certainly induce too much tone on the uterus, and thereby exclude the benefit of turning, which can only arise from a uterus of moderate tone, bordering on relaxation. By fixing my finger in the groins of the child, and by strong lateral motion, I at length extracted the lower extremities, and terminated the labour successfully; but I am very confident, that under circumstances of defective stimulation from the fatigue the woman had endured, and the inutility of some hours pains, that the case must otherwise have come to a turning.

The child, on its birth, did not assume the respiratory function, so that we all thought it was what is termed still born, and as it was much longer than usual we were a little surprized when the respiration commenced. I shall leave more able physiologists to determine what relation the spina bifida bore to this circumstance, as I do not wish to occupy too much of your valuable though limited pages.

The tumour, on the first day, resembled a bag or bladder, half filled with fluid, excessively thin and diaphanous, so that it appeared a most simple substance either epidermial or membranous; but though the organism of this covering was so fine as to elude the passage of red blood, yet it appeared to retain much excitability, and accommodate itself to the circulation of the infant, by which day after day it approximated more and more to the nature of the cutis vera. On the first day, by holding a strong light on one side, and viewing it on the opposite, I could not discover the ramifications of the vessels. The next day the red blood was forced into them, presenting the most exquisite vascularity, and the most beautiful retiform distribution I ever beheld. The diameter of the tumour was increased as well as the excitement, and thus it proceeded till it arrived at the appearance depicted in the drawing. Ulcerative inflammation at last commenced, and about the eighteenth day from the birth, the child died from the rupture of the cyst and evacuation of its contents.

The lower extremities, though in so infantine a state as our patient, yet bore a strong resemblance to the palsy of the lower limbs, described by Mr. Pott as arising from a scrophulous disease in the vertebræ, and though the causes of the two diseases may vary, their effects may be analogous on the spiral marrow; but why might not the same cause operate on the ossification of the vertebræ of a child *in utero*, and therefore be attended with analogous effects? for the infant never used the lower extremities, and they evidently wasted more than the rest of the body.

The

The drawing represents the tumour in the middle stage of its progress, inflamed, red, thickened, hot, excessively vascular, and scarcely transparent, with incipient ulceration in its outer surface, attended with slight oozing between the lamina of the membrane, or otherwise by secreting surface. It ought to be noticed that the skin ascended on the tumour in narrow lines, though very partially; whether therefore it imparted a similar structure to the rest of the tumour, is a question.

## Figures and Tables

**Figure f1:**